# Smallpox in Valencia, Spain

**Published:** 1902-03

**Authors:** 


### Smallpox in Valencia, Spain.

The Alcalde of Valencia has issued the following proclamation: 1. Vaccination and revaccination of all inhabitants not already vaccinated is declared compulsory in Valencia. 2. To facilitate compliance with this order, without expense or trouble to the inhabitants, all sanitary posts and relief stations shall be permanently open. 3. All municipal doctors shall make known to the sick poor that they can only assist and supply medicines gratuitously on condition that patients and their families be vaccinated within twenty-four hours. 4. All school masters, both public and private schools, shall require certificates of vaccination from pupils. 5. Laborers employed by the municipality will not receive wages unless they present medical certificates of vaccination. 6. Directors and owners of factories and workmen of all classes shall exact from all employes certificates of vaccination under penalty of fine. 7. Directors of penitentiaries, hospitals, almshouses, homes for invalids, asylums, sanatoria, or similar establishments, shall proceed immediately to vaccinate or revaccinate all those under their care, and refuse admittance to visitors not furnished with vaccination certificates. 8. In the shelter sheds and casualty wards for paupers no admission or assistance will be given to the unvaccinated unless they submit to vaccination on the spot. 9. Charity tickets distributed on feast days by submayors will not be honored unless accompanied by certificates of vaccination. 10. All applications for stalls and booths in public markets must be accompanied by such certificates. 11. No name of any unvaccinated person shall be inscribed on the parish poor list. 12. Submayors shall visit and inspect all houses in their respective districts, beginning with those in which cases of smallpox exist, and shall insist upon the immediate vaccination of all who have not already complied with this order. 13. Proprietors of hotels and lodging houses shall refuse to admit anyone without proof of recent vaccination. The infraction of any of the foregoing articles will be punished with a fine of fifty pesetas (about $10.00).—*Philadelphia Medical Journal.*

[Texas can here learn a lesson from poor old Spain. Texas should enact such a law without delay.—Ed.]

